# Delayed vitreous prolapse after cataract surgery: clinical features and surgical outcomes

**DOI:** 10.1038/s41598-021-95527-0

**Published:** 2021-08-09

**Authors:** Tae Young Kim, Hyun Goo Kang, Chan Yun Kim, Hyoung Jun Koh, Sung Soo Kim, Min Kim

**Affiliations:** 1 Department of Ophthalmology, Institute of Vision Research, Gangnam Severance Hospital, Yonsei University College of Medicine, 211 Eonjuro, Gangnam-gu, Seoul, 06273 Korea; 2 Department of Ophthalmology, Institute of Vision Research, Severance Eye Hospital, Yonsei University College of Medicine, 50-1 Yonseiro, Seodaemun-gu, Seoul, 03722 Korea

**Keywords:** Eye manifestations, Ocular hypertension, Retinal diseases, Vision disorders, Risk factors

## Abstract

This study investigates the etiology and clinical features of delayed vitreous prolapse after cataract surgery and evaluates the long-term surgical and visual outcomes. Consecutive patients with vitreous prolapse into the anterior chamber occurring ≥ 3 months after cataract surgery at two hospitals between December 2006 and June 2020 were retrospectively reviewed. The primary outcome was associated ophthalmological events that triggered delayed vitreous prolapse. Secondary outcomes included long-term visual and subjective symptom changes after treatment. Among 20 eyes (20 patients), all had visual symptoms, the most common being blurry vision (12 patients; 60%). Five (25%) were detected after YAG laser capsulotomy, three (15%) had a history of intraocular lens(IOL) implantation in sulcus due to intraoperative posterior capsular tears, three (15%) had prolapsed vitreous alongside dislocated IOLs, and three (15%) were aphakic after previous cataract surgeries. After surgical treatment, the mean corrected distance visual acuity improved from 20/50 to 20/31(*P* = 0.02) and the mean preoperative intraocular pressure (IOP) that was 26.4 mmHg decreased to 15.6 mmHg, remaining stable until the last follow-up. All reported symptoms were relieved. YAG laser capsulotomy or a history of defective posterior capsule from iatrogenic causes may trigger delayed vitreous prolapse. The long-term outcomes were favorable, particularly after posterior vitrectomy, with improved IOP control and symptom resolution.

## Introduction

Vitreous prolapse or loss is a condition in which the vitreous gel becomes displaced beyond the posterior capsule into the anterior segment of the eye. Previous reports have mentioned that the incidence of vitreous loss ranges from 1.4 to 3.2% during cataract surgery, and 7.65% by learning surgeons but no studies have focused on the prevalence of delayed vitreous loss long after the primary surgery^1–3^. Additionally, few case reports have documented possible ophthalmological complications of vitreous prolapse, such as pupillary block glaucoma with intraocular pressure (IOP) elevation^4,5^, or corneal decompensation leading to bullous keratopathy, cystoid macular edema, uveitis, or even retinal detachment^6,7^.

The possible risk factors for vitreous loss during surgery are age < 40 years and pseudoexfoliation^2^, while case reports have documented that Nd:YAG laser capsulotomy for posterior capsular opacification may induce vitreous prolapse in pseudophakic eyes^4,5^. However, most case series have focused on intraoperative vitreous prolapse, and therefore, little is known about the clinical characteristics, demographics, and outcomes of vitreous prolapse occurring long after the primary surgery. Only the individual case reports have been published. In addition, the treatment outcomes of delayed vitreous prolapse, in terms of the visual outcomes, complications, and subjective symptoms, are either poorly documented or outdated in the age of microincisional phacoemulsification^8^.

In this study, we investigated the possible etiology, demographics, and clinical features of delayed vitreous prolapse after cataract surgery. We also evaluated the long-term surgical and visual outcomes.

## Materials and methods

This was a retrospective study of patients diagnosed with vitreous prolapse between December 2006 and June 2020 at two high-volume, referral-based tertiary hospitals: Severance Eye Hospital and Gangnam Severance Hospital, affiliated with Yonsei University College of Medicine. Institutional Review Board (IRB) approval was obtained from Gangnam Severance Hospital IRB (No 3-2020-0371), and the study adhered to the tenets of the Declaration of Helsinki. The requirement for informed consent was waived by the Gangnam Severance Hospital IRB due to the retrospective nature of the study with no identifying feature or figure that requires additional patient consent.

### Study selection

We included patients with vitreous prolapse into the anterior chamber occurring at least 3 months after the initial surgery. After a careful review of the electronic medical records of all included cases, we excluded all patients with an underlying ophthalmological anatomical or trauma event that occurred within the 3 months before the diagnosis of vitreous prolapse. Thus, we excluded patients who suffered vitreous loss after an intraoperative posterior capsule rupture, during any other surgical procedure, or immediately after an ocular traumatic event.

### Data collection

All patient demographics, clinical, and ocular features were evaluated using electronic records or imaging data. The patients’ slit photo images were also assessed. Visual acuity (VA) was measured using a Snellen chart and converted to the logarithm of the minimum angle of resolution (logMAR) for statistical purposes. IOP was mainly measured using a Goldmann applanation tonometer and/or a rebound tonometer (iCare; Revenio, Vantaa, Finland). Endothelial cell counts (cells/mm^2^) were measured using specular microscopy (Konan Noncon Robo SP8000 or Konan NonCon Cellchek XL NSP-9000; Konan Medical Inc., Hyogo, Japan), while axial lengths (mm) were measured using either the IOL-master 600 or IOL-master 700 (Zeiss Meditec AG, Jena, Germany). Central macular thickness (CMT) and the presence of intraretinal fluid (IRF) or subretinal fluid (SRF) were analyzed using optical coherence tomography (OCT; Heidelberg Engineering, Heidelberg, Germany). Patient symptoms were retrieved from the electronic documents and checked for subjective improvement at each follow-up visit.

### Surgical technique

Surgery was performed by experienced retinal specialists (M.K. and H.J.K). When complete vitrectomy was deemed necessary by the attending surgeon, a standard three-port vitrectomy was performed using a 25-gauge system (Constellation; Alcon Laboratories, Fort Worth, TX, USA). Anterior vitrectomy was performed using the Centurion Vision System (Alcon, Fort Worth, Texas, USA). All surgeons used triamcinolone acetonide (Maqaid, Wakamoto Pharmaceuticla Co., Ltd., Tokyo, Japan) for the visualization of vitreous in anterior and posterior segment and to completely remove vitreous during the vitrectomy procedure.

### Main outcome measures

The primary outcome was associated ophthalmological events that triggered delayed vitreous prolapse. Secondary outcomes included long-term corrected distance visual acuity (CDVA), intraocular pressure, and subjective visual symptom changes after treatment.

### Statistical analysis

We analyzed the data using SPSS software version 25.0 (IBM Corp., Armonk, NY, USA). Variables such as VA and IOP were compared between groups set according to post-operative time periods using two-sample paired t-tests after testing for normality using the Kolmogorov–Smirnov test. A *P*-value of < 0.05 was considered to be statistically significant.

## Results

Among 199 cases with vitreous prolapse, 179 with prolapse occurring intraoperatively or immediately following cataract surgery were excluded, and twenty eyes from 20 (10%) patients with delayed prolapse who met the inclusion criteria were included. The baseline characteristics are summarized in Table 1. The mean age was 60 years (median: 59 years, range: 43–82 years), with a male preponderance (16 males; 80%). The mean follow-up duration was 48 months (median: 30 months, range: 1–143 months) after the diagnosis of vitreous prolapse. The mean duration from cataract surgery to vitreous prolapse was 94 months, ranging from 3 to 498 months (> 40 years).

**Table 1:** Baseline characteristics for patients with delayed vitreous prolapse.

Baseline characteristics (n = 20, 20 eyes)	
Age, years	60.20 ± 10.68 (43–82)
**Gender, n (%)**	
Male	16 (80.0%)
Female	4 (20.0%)
Hypertension, n (%)	7 (35.0%)
Diabetes, n (%)	0 (0.0%)
**Visual Symptoms**	20 (100.0%)
Blurring	12 (60.0%)
Decrease in vision	4 (20.0%)
Floaters	2 (10.0%)
Flashes	1 (5.0%)
Injection	1 (5.0%)
Duration from cataract surgery to prolapse (months)	94.3 ± 136.2 (median: 38.6, 3.4–498.1)
**Visual acuity, logMAR (Snellen equivalent)**	
At first visit	0.41 ± 0.46 (20/50)
At last visit	**0.19 ± 0.25 (20/31)**
**Intraocular pressure (mmHg)**	
Pre-operation	26.35 ± 9.12 (14–42)
Post-operation	**15.57 ± 2.80 (11–21)**
Pre-operative endothelial cell count (cells/mm^2^)	2042.58 (529–2976)
Axial length (mm)	24.14 ± 0.51 (22.82–25.08)
Follow up duration (months)	48.37 (1–143)

Age, visual acuity, and intraocular pressure displayed as mean ± SD.

N displayed as number (%).

Follow-up duration = post-operative follow-up period after surgical intervention for the prolapse.

FC = 2.00 logMAR, HM = 2.30 logMAR, LP = 2.60 logMAR, no light perception = 2.90 logMAR.

*logMAR* logarithm of the minimal angle of resolution.

All 20 patients had visual symptoms, the most common being blurry vision (12 patients; 60%), followed by decreased VA (four patients; 20%). The mean initial VA at the first visit was logMAR 0.41 (Snellen equivalent: 20/50, range: 20/500 to 20/20), which had improved to logMAR 0.19 (Snellen equivalent: 20/31, range: 20/125 to 20/20) at the patients’ most recent visit. The mean IOP before treatment for vitreous prolapse was 26.35 mmHg (median: 26 mmHg, range: 14–42 mmHg), with 11 cases (55%) of elevated IOP (> 22 mmHg), and a mean endothelial count of 2042 cells/mm^2^ (median: 2227 cells/mm^2^, range: 529–2976 cells/mm^2^). All 11 patients had acute IOP elevation along with visual symptoms when the slit lamp examination first revealed vitreous loss. The mean axial length was 24.14 mm (median: 24.2 mm, range: 22.82–25.08 mm). Surgical treatment was performed a mean of 119 days after the diagnosis of vitreous loss (median: 9 days, range: 3–1318 days), and no new severe complications occurred during this period.

### Previous surgical history prior to detection of vitreous loss

The patients’ previous ophthalmological history before the diagnosis of delayed vitreous prolapse are summarized in Table 2. Five patients (25%) had symptoms manifest after YAG laser capsulotomy, three (15%) had a previous surgical history of intraocular lens (IOL) implantation in the ciliary sulcus due to intraoperative posterior capsular tears, three (15%) had prolapsed vitreous alongside IOL dislocation, and three (15%) were aphakic after previous cataract surgeries performed in the 1980s. Those patients presenting with dislocated IOLs did not have a history of ocular trauma, or records indicating complications during cataract surgery, such as capsular ruptures or in-sulcus IOL placement. The cause of vitreous prolapse was unknown in six pseudophakic patients (30%) with seemingly intact posterior capsules and no identifiable zonular weakness or pseudophacodonesis. Gangnam Severance Hospital and Severance Eye Hospital had 70 cases (2.5%) of posterior capsular tear among 2775 cataract surgeries in 2019. During our study period, three cases (1.5%) among 199 cases of vitreous prolapse met our study’s inclusion criteria of delayed prolapse and were caused by posterior capsular tear which is a rare incidence.

**Table 2:** Patient factors and treatment of vitreous prolapse.

Patient factors and treatment (n = 20, 20 eyes)	
**Previous ophthalmologic history**	
Nd: YAG laser, n (%)	5 (25.0%)
IOL in sulcus + posterior capsular tear during surgery, n (%)	3 (15.0%)
IOL dislocation, n (%)	3 (15.0%)
Aphakia, n (%)	3 (15.0%)
None (unknown cause)	6 (30.0%)
Duration from prolapse to surgical treatment (days)	118.6 (median: 17, 3–1318)
**Ocular comorbidity**	
Glaucoma	7(35.0%)
Primary open angle glaucoma	5 (25.0%)
Secondary glaucoma (d/t uveitis)	2 (10.0%)
Central retinal vein occlusion	1 (5.0%)
None	12 (60.0%)
**Treatment for vitreous removal**	
Anterior vitrectomy, n (%)	13 (65.0%)
Trans pars plana vitrectomy, n (%)	7 (35.0%)
Relapse rate	0%
Post-operative remnant vitreous	1 (5.0%)

Age, visual acuity, intraocular pressure displayed as mean ± SD

N displayed as number (%).

*logMAR* logarithm of the minimal angle of resolution, *IOL* intraocular lens, *Nd:YAG* neodymium-doped yttrium aluminum garnet.

In terms of past ophthalmic history, seven patients had a history of glaucoma, among whom five (25%) were diagnosed with primary open angle glaucoma and two (10%) with secondary glaucoma due to uveitis; one patient had a history of central retinal vein occlusion. Finally, the rest (12 patients; 60%) had no other history of ocular comorbidities. Slit lamp examination revealed no pseudoexfoliation in seven patients with the history of glaucoma.

### Treatment for vitreous prolapse

Surgical treatment was performed in all 20 patients: 13 (65%) received anterior vitrectomy and seven (35%) underwent posterior pars plana vitrectomy. One patient underwent anterior vitrectomy for remnant vitreous in the chamber identified immediately after surgery, while all other cases showed no relapse of vitreous loss. Three patients had vitreous loss alongside dislocated (IOL): two received simple IOL repositioning and one had his IOL exchanged.

### Long-term visual outcomes and complications

Overall, vision improved significantly after vitreous removal, with the mean post-operative corrected distance VA (CDVA) improving to logMAR 0.26 ± 0.29 (Snellen equivalent: 20/37, range: 20/200 to 20/20; *P* = 0.042) at 1 month. This improvement was maintained until the patients’ last visit, at which the mean logMAR was 0.20 (Snellen equivalent: 20/31, range: 20/125 to 20/20; *P* = 0.02).

**Figure 1:**
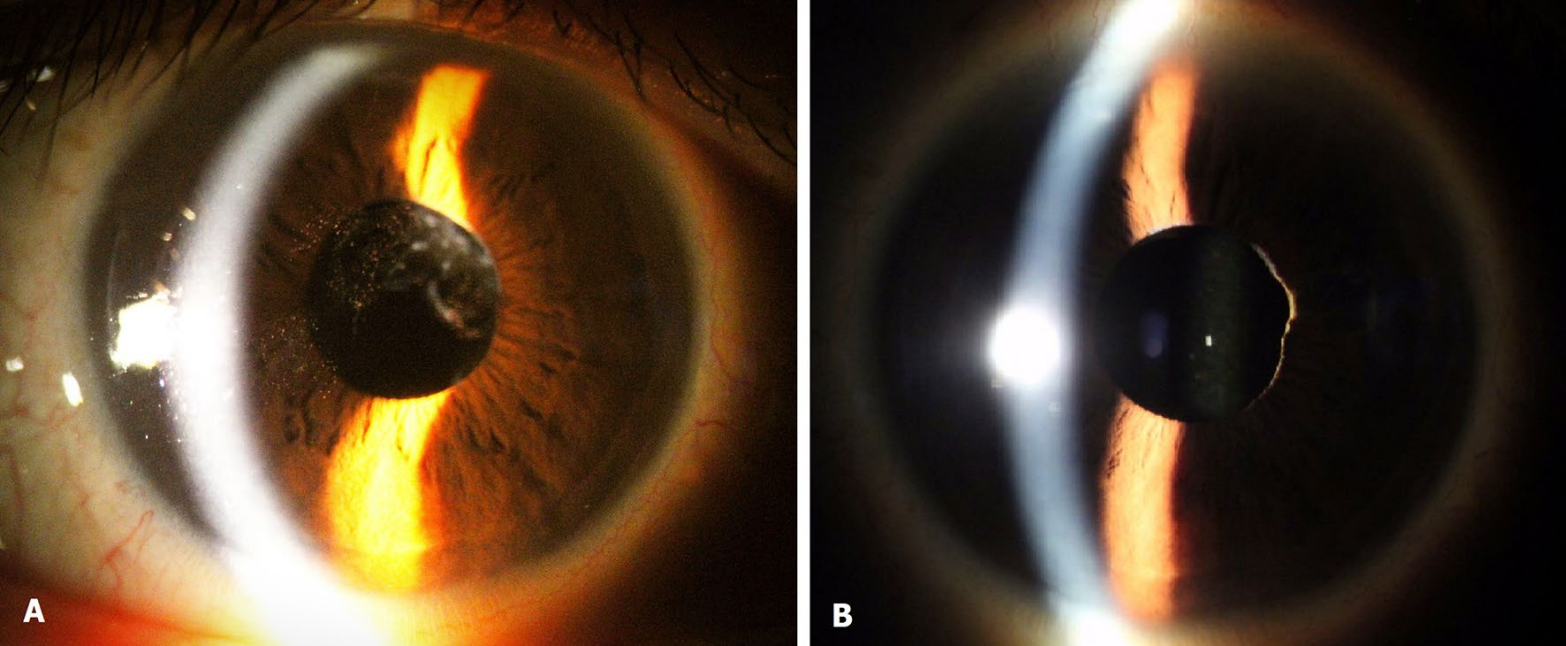
Pre-operative and post-operative external photograph of an eye with vitreous prolapse. The patient was a 55-year-old male who presented with a chief complaint of flashes and increased intraocular pressure of 37.9 mmHg in his right eye. **(A)** Vitreous prolapse at initial presentation. Pigment cells were observed in the anterior chamber vitreous, but dilated fundus examination revealed no sign of retinal break or detachment. **(B)** Successfully removed vitreous prolapse after trans pars plana vitrectomy. After the surgical treatment, IOP decreased from 37.9 to 11 mmHg with improved visual discomfort. The notch in the patient’s nasal iris was present before the surgical removal of vitreous.

The mean IOP had decreased to 15.7 ± 3.9 mmHg (*P* < 0.001) 1 month after surgery, and 15.0 ± 2.3 (*P* = 0.002) after 6 months. The IOP remained stable at 15.6 ± 2.8 mmHg (*P* < 0.001) until the patients’ last visit. After surgery, seven of the 11 patients (63.6%) with elevated IOP had at least one of their anti-glaucoma eye drops discontinued. The same patients also ceased oral intake of IOP-lowering systemic carbonic anhydrase inhibitors. We tracked the visual symptoms of all 20 patients summarized in Table 1. The five symptoms which were blurring, vision decrease, floaters, injection, and flashes, had disappeared 1 month after surgery. The visual and IOP outcomes are listed in Table 3, along with representative images from before and after the removal of prolapsed vitreous in Fig. 1.

**Table 3:** Outcomes in patients after removal of vitreous prolapse.

Outcomes in patients after removal of vitreous prolapse (n = 20)						
	Vision	*P*-value	IOP	*P*-value		Symptoms (n = 20)
Initial	0.41 ± 0.46 (20/50)		26.4 ± 9.1 (14–42)	–		Blurring: 12 Vision decrease: 4 Floater: 2 Injection: 1 Flash: 1
1 month post-op	0.26 ± 0.29 (20/37)	0.042*	15.7 ± 3.9	< 0.001*		Blurring: 0 Vision decrease: 0 Floater: 0 Injection: 0 Flash: 0
6 months post-op	0.29 ± 0.37 (20/39)	0.058	15.0 ± 2.3	0.002*		Blurring: 0 Vision decrease: 0 Floater: 0 Injection: 0 Flash: 0
Last visit	0.19 ± 0.25 (20/31)	0.017*	15.6 ± 2.8	< 0.001*		Blurring: 0 Vision decrease: 0 Floater: 0 Injection: 0 Flash: 0

Average post-operative follow-up duration: 48 months.

Values displayed as mean ± SD.

Visual outcomes compared to initial visual acuity.

Two-sample paired *t* tests. A *P*-value less than 0.05 considered statistically significant.

Normality test done using the Kolmogorov–Smirnov test. All groups followed normal distribution (*P* > 0.05).

FC = 2.00 logMAR, HM = 2.30 logMAR, LP = 2.60 logMAR, no light perception = 2.90.

*IOP* intraocular pressure.

We additionally checked the CMT and the presence of IRF or SRF using OCT of patients with vitreous prolapse and no patients showed any signs of macular edema or retinal fluid. The mean central macular thickness was 267.2 ± 39.0 μm (median 273.4 μm, range: 198 to 319). We re-analyzed the OCT of patient with CMT of more than 300 μm and no overt IRF, SRF, or cystic change was found, which could be deemed as a subclinical thickening of central macula.

In one patient, we assessed endothelial cell count before and after vitreous prolapse. The cell count decreased from 2280 cells/mm^2^ 8 months before the prolapse to 1470 cells/mm^2^ 8 months after the detection. This patient received anterior vitrectomy to remove the prolapse. We identified one patient with severe complications leading to low vision and bullous keratopathy requiring penetrating keratoplasty. However, this particular patient already had a pre-surgical specular count of 800 cells/mm^2^ and had been warned of the risk of corneal decompensation after surgery.

## Discussion

In this study, we retrospectively reviewed patients with delayed vitreous prolapse occurring after intraocular surgery or a traumatic ocular event, documenting the clinical and demographic characteristics and long-term outcomes in these patients. In particular, over a quarter of our patients experienced symptomatic vitreous loss after YAG laser capsulotomy, and over 15% had a prior history of intraoperative posterior capsular tear necessitating IOL implantation in the ciliary sulcus. Both of these factors may have led to the delayed prolapse of vitreous gel into the anterior chamber. Vitreous prolapse caused various visual discomfort (reduced VA, floaters, flashes), and over 50% of patients suffered from IOP elevation requiring numerous anti-glaucoma medications. Surgical treatment resulted in significant and sustained improvement of vision, symptoms, and IOP, which was evident from the first month after surgery and lasted until the most recent visit after a mean follow-up of 48 months. One patient with already poor endothelial count experienced unfavorable outcomes, while the rest experienced no complications.

Vitreous prolapse is a surgical complication of cataract surgery that can induce macular edema, corneal decompensation, glaucoma, and retinal detachment. The incidence rates vary among different studies from 1.7 to 7.7% depending on the surgeon’s experience^9^. Occasional case reports have mentioned that delayed vitreous prolapse is likely caused by Nd:YAG laser capsulotomy. The authors of one such report postulated that lasers disrupt the anterior vitreous face, causing vitreous prolapse into anterior segment^10^. When performing Nd:YAG laser capsulotomy, surgeons should not extend the margin beyond the edge of the optic to avoid vitreous herniation and loss around the optic^11^. Minimizing energy by targeting the appropriate central optical zone is crucial to prevent vitreous prolapse.

Some studies have mentioned the risk factors that contribute to an increased likelihood of vitreous loss during phacoemulsification. They include deep-set eyes, narrow palpebral fissures, high myopia, and previous history of vitreous loss^12^. Our patients with delayed vitreous prolapse had an axial length of 24.09 ± 0.55 mm, which is not in the range of high myopic axial length generally defined as longer than 26.0 mm and spherical equivalent of -6D or worse^13^. This indicates that the risk factors for delayed vitreous loss differ from those for intraoperative vitreous prolapse.

The subjective symptoms experienced by these patients are poorly reported. In our own series, we found that patients presented with visual symptoms, ranging from blurred vision (57.1%) and VA decrease (23.8%) to flashes of light. Thus, even simple symptoms that may indicate posterior vitreous detachment or floaters should not be overlooked. Ophthalmologists should perform diligent anterior segment evaluations using slit lamp microscopy to detect delayed vitreous loss, especially in patients with a history of intraoperative posterior capsule rupture or recent YAG laser capsulotomy.

Overall visual prognosis was favorable after the vitreous prolapse was treated. All 20 patients underwent surgical removal of the prolapsed vitreous via either anterior vitrectomy (62.0%) or pars plana vitrectomy (38.0%); none of the patients showed recurrent vitreous prolapse. Although not every patient achieved perfect CDVA recovery of 20/20 or more due to their ocular comorbidity and history of glaucoma, uveitis, ECC reduction or CRVO after the surgical treatment, the CDVA always improved along with symptoms resolution and IOP reduction. The patients’ elevated IOP also significantly decreased to normal IOP ranges after vitreous removal. In terms of anterior versus pars plana vitrectomy, Ryoo et al. showed that 23-gauge pars plana vitrectomy had less post-operative complications than anterior vitrectomy when removing prolapsed vitreous during cataract surgery^14^. Our current study also implied that pars plana vitrectomy is beneficial for vitreous removal in the long-term, as one patient had remnant vitreous in anterior chamber after anterior vitrectomy. For the case of remnant vitreous after initially receiving anterior vitrectomy, the surgeon confirmed the complete removal of vitreous by triamcinolone staining and pupil constriction as is the standard protocol at our centers; however, the recurrence of vitreous prolapse in the immediate postoperative period may indicate that there was (1) vitreous concealed in the posterior chamber, or (2) loose vitreous that remained trapped in the vitreous cavity due to gravity during surgery, but subsequently may have protruded again by ocular movement right afterwards. Altogether, this case highlights the need for careful and thorough evaluation of the posterior chamber and peripheral iris, and may indicate that performing pars plana vitrectomy for more complete removal of the vitreous gel would be advantageous in these complication cases. Since pars plana vitrectomy allows more complete and thorough vitrectomy, and it may be more reliable. Moreover, performing the pars plana vitrectomy allows additional benefit of managing the well-recognized complication of vitreous prolapse like retinal tear.

With regard to the quality of life after the procedure, surgical treatment of vitreous prolapse alleviated all visual discomfort, without any aggravation, until the patients’ last visit. Considering that vitreous loss can induce considerable ocular complications, surgeons should consider surgical removal of prolapsed vitreous once a diagnosis is made, even if the condition does not immediately harm the patient.

This study was limited because it was retrospective, and therefore, we could not control all the baseline characteristics. Furthermore, partly due to the study design, we could not closely analyze the change in endothelial cell count, which may have been affected by vitreous touching the corneal endothelium. Patients with delayed prolapse were usually referred to our institution by other clinics, and therefore data regarding their preoperative endothelial cell density counts were unavailable for analysis. In addition, we were unable to find a definite cause of prolapse in six patients (30.0%). However, the strength of the study was that we presented a series of patients with a rare surgical complication. This allowed us to suggest possible etiologies for the delay in vitreous loss, present possible risk factors, and document both the clinical spectrum and long-term treatment outcomes. Further clinical studies focusing on the causal relationship between different factors and delayed vitreous prolapse are necessary to better understand this rare complication.

In conclusion, delayed vitreous prolapse after cataract surgery can be caused by YAG laser capsulotomy or previous surgical history of posterior capsular tear. The long-term anatomical and visual outcomes of removing vitreous prolapse are favorable, especially in the case of pars plana vitrectomy, with improved visual acuity, IOP control and reduced symptoms. Ophthalmologists should be aware of the possibility of vitreous prolapse when performing YAG laser capsulotomy and should actively treat once the prolapse is noted.

## Data Availability

The datasets generated during and/or analysed during the current study are not publicly available due to privacy laws and policies in Korea, but are available from the corresponding author on reasonable request.
